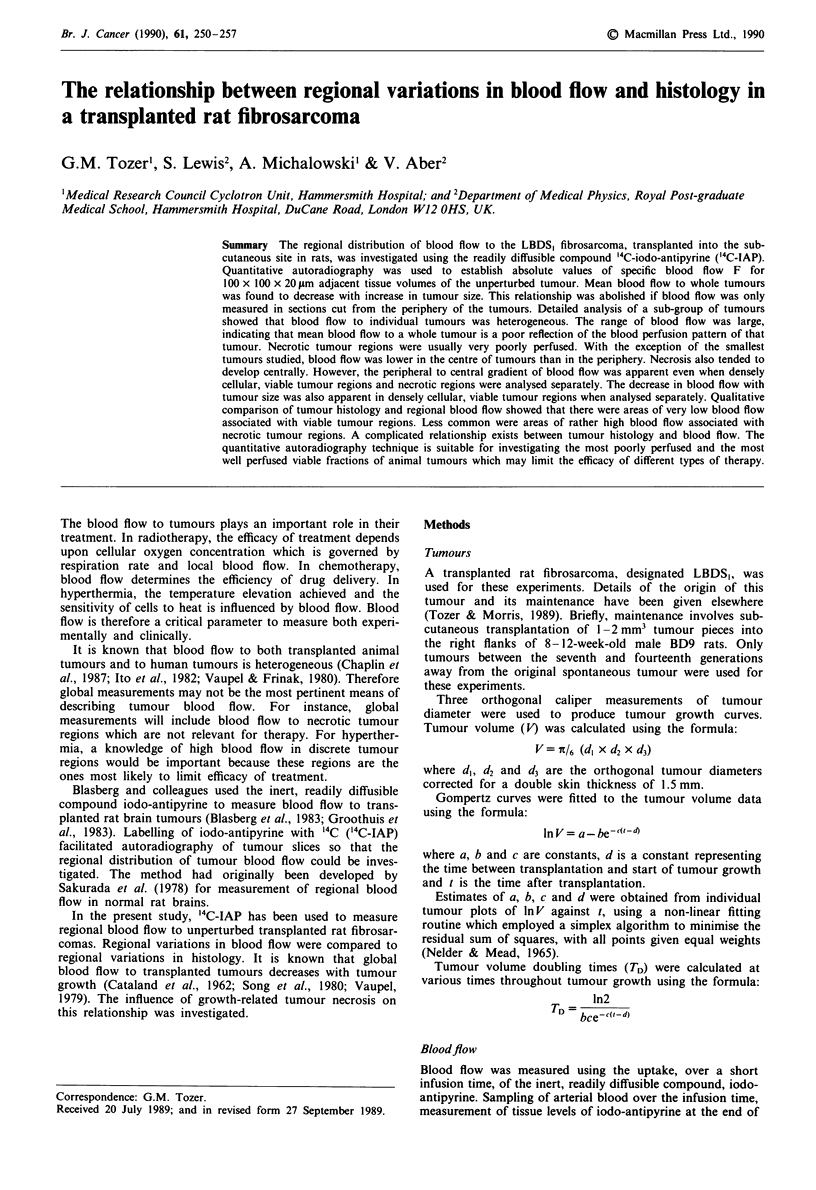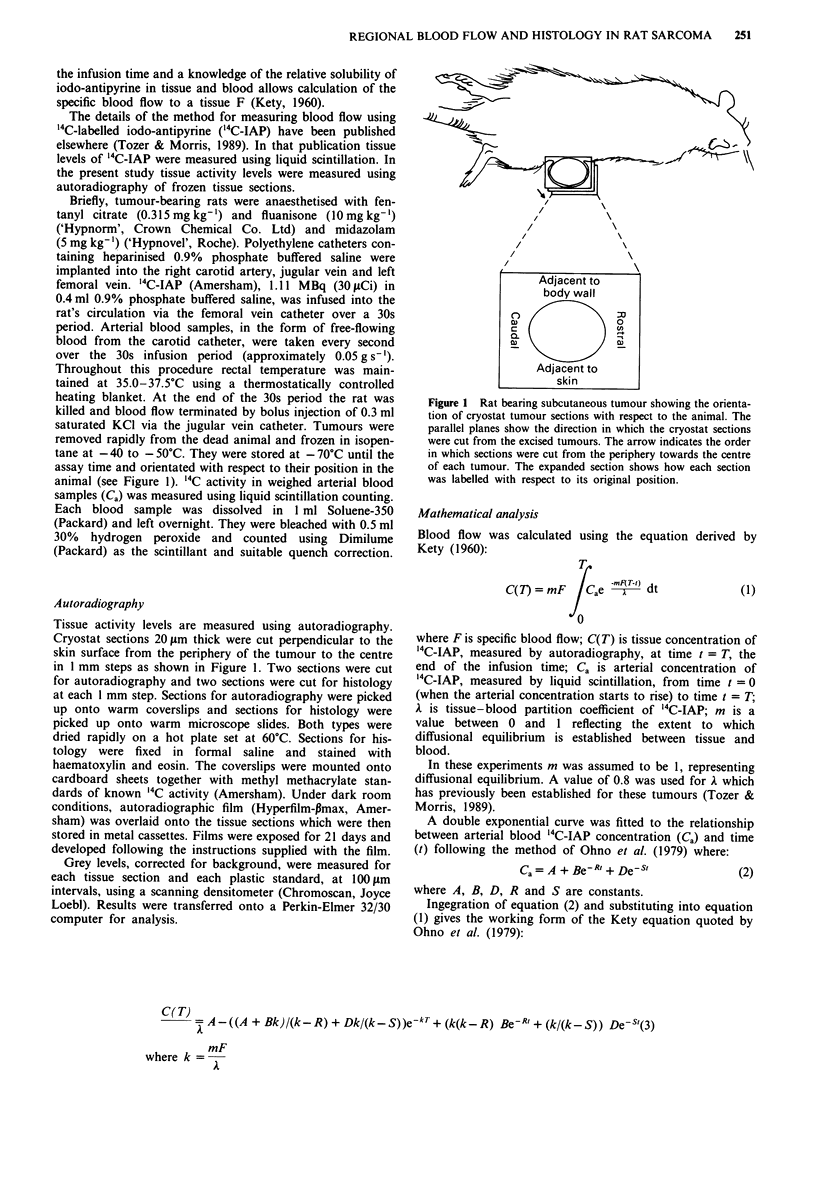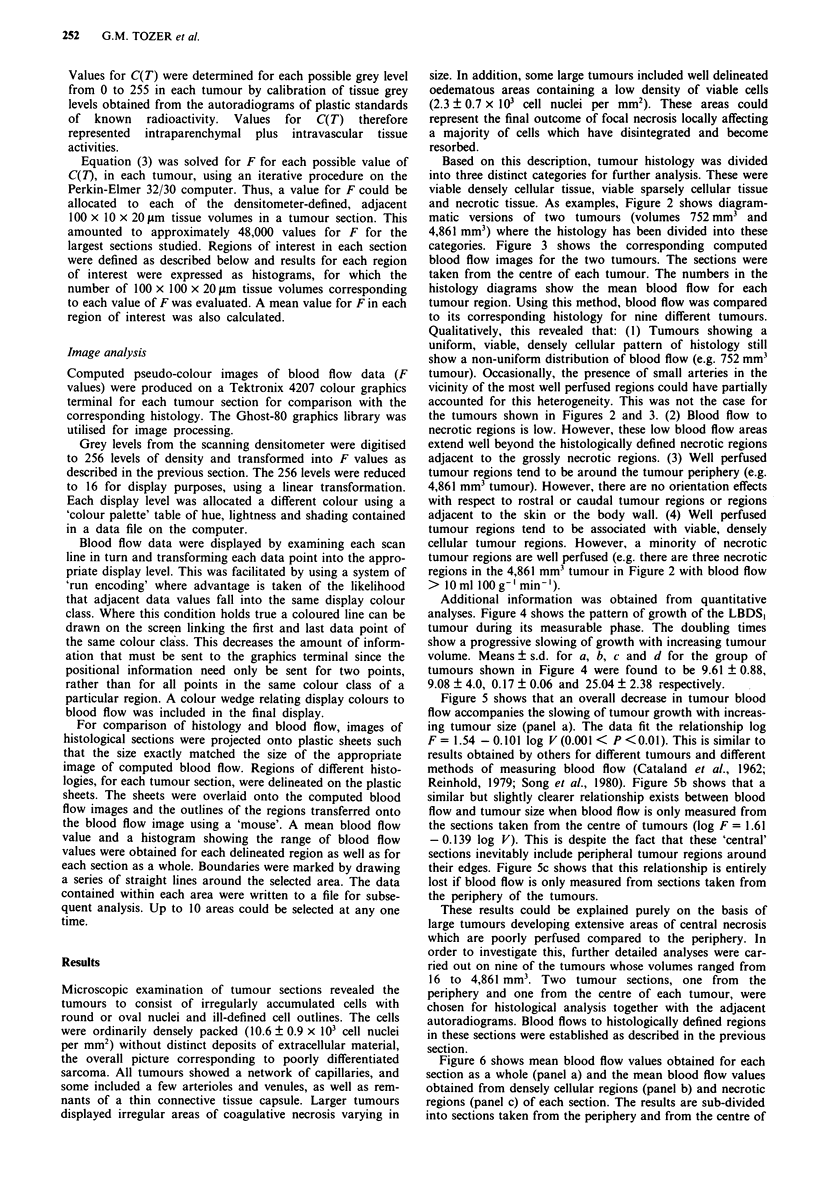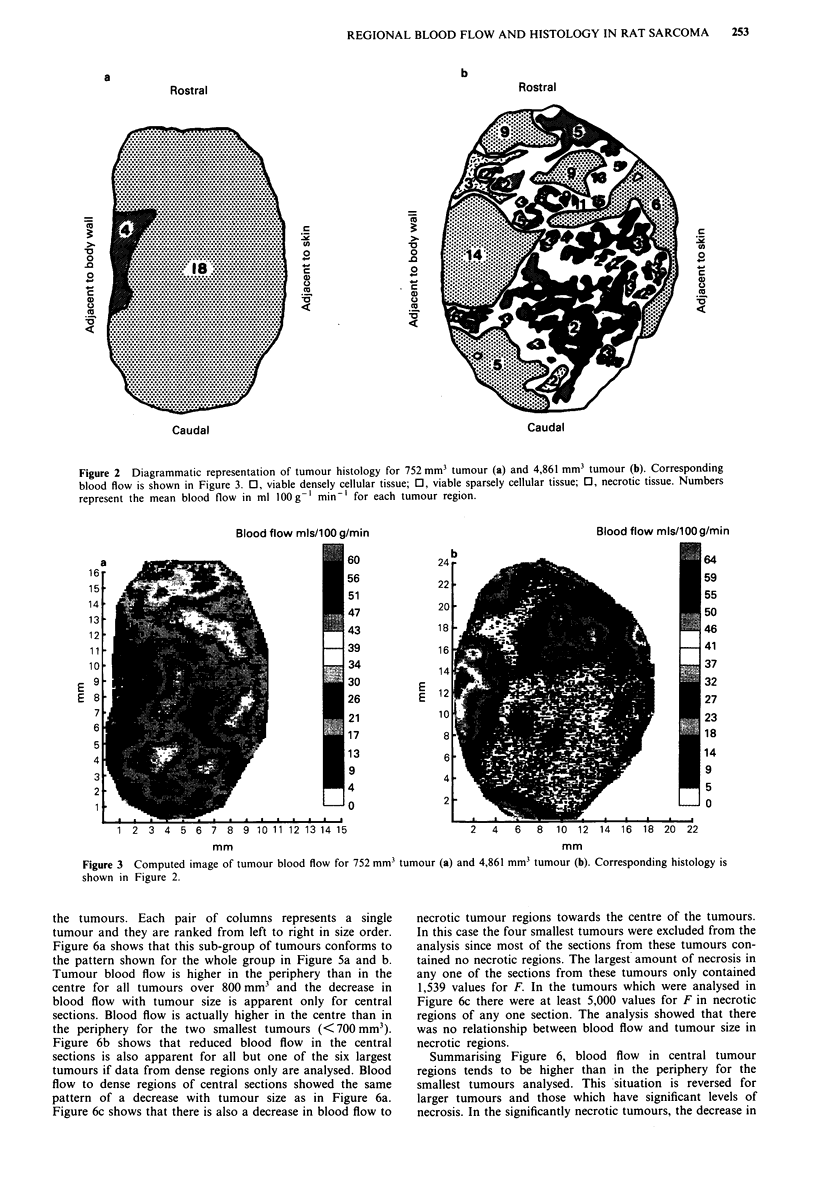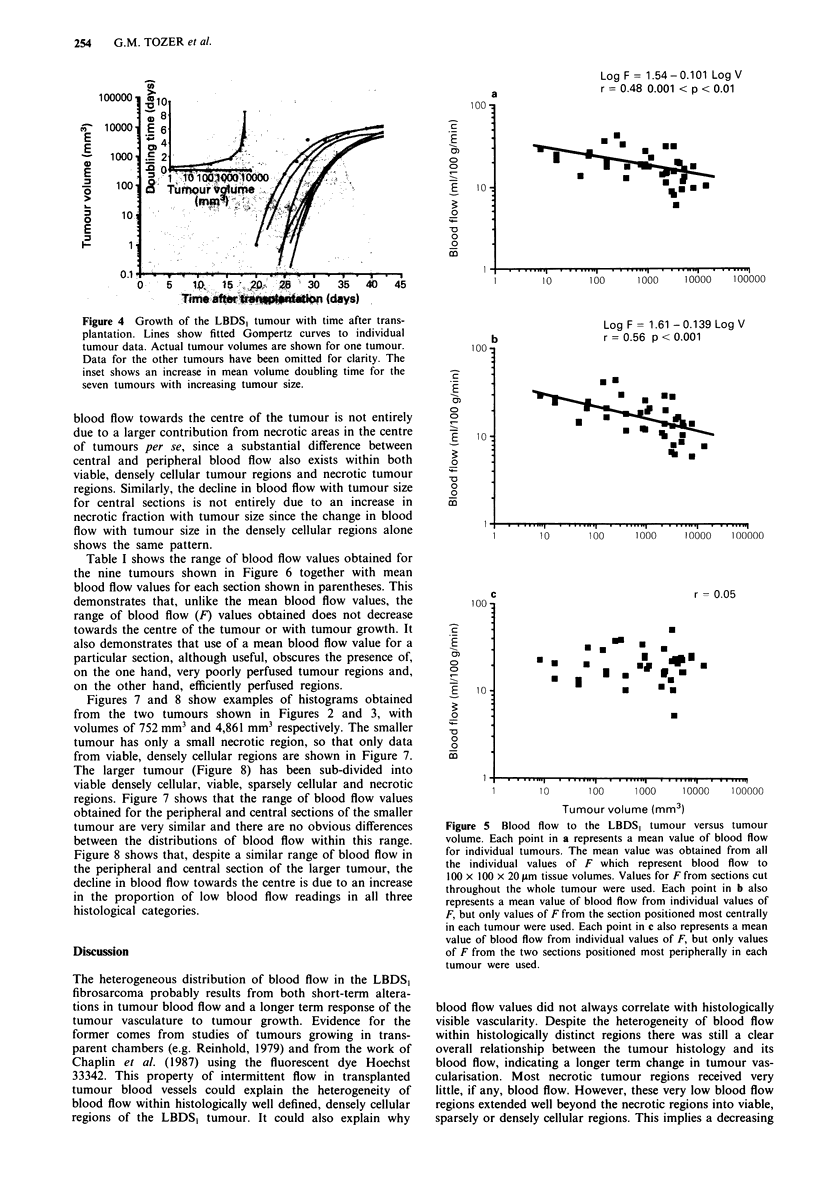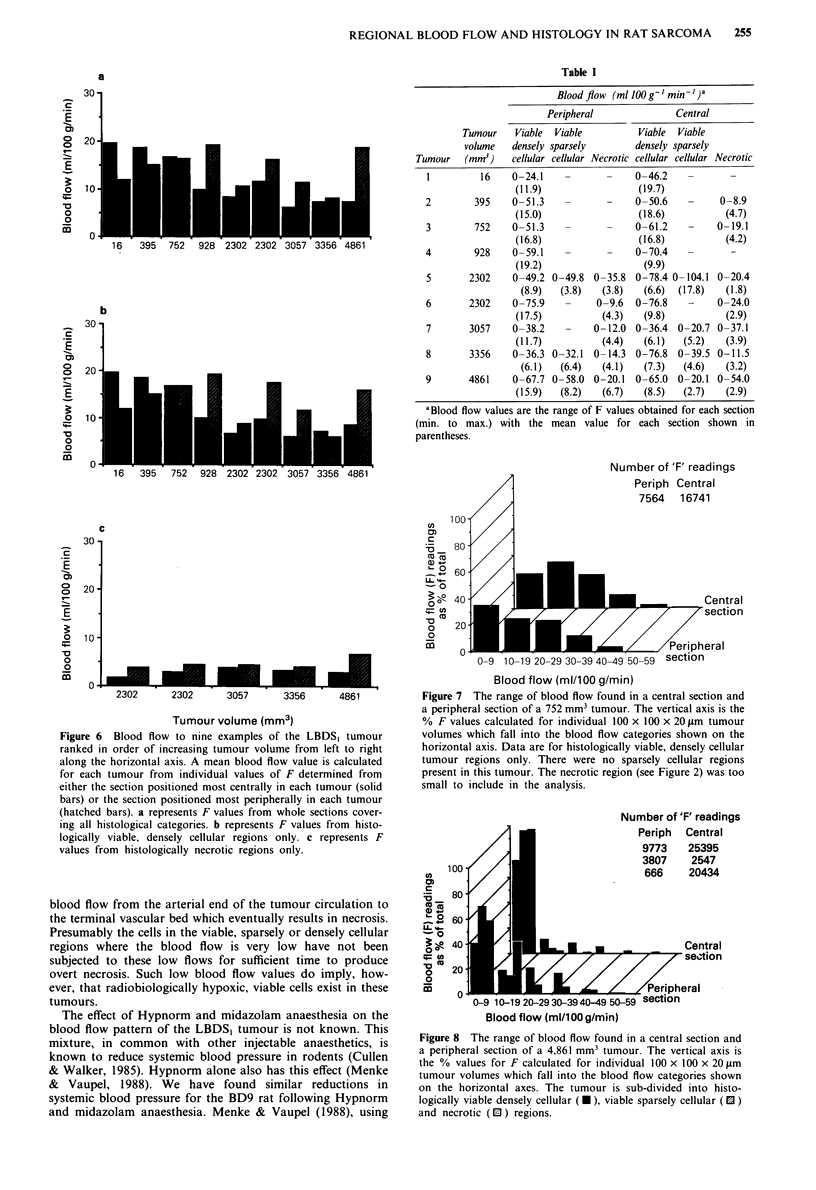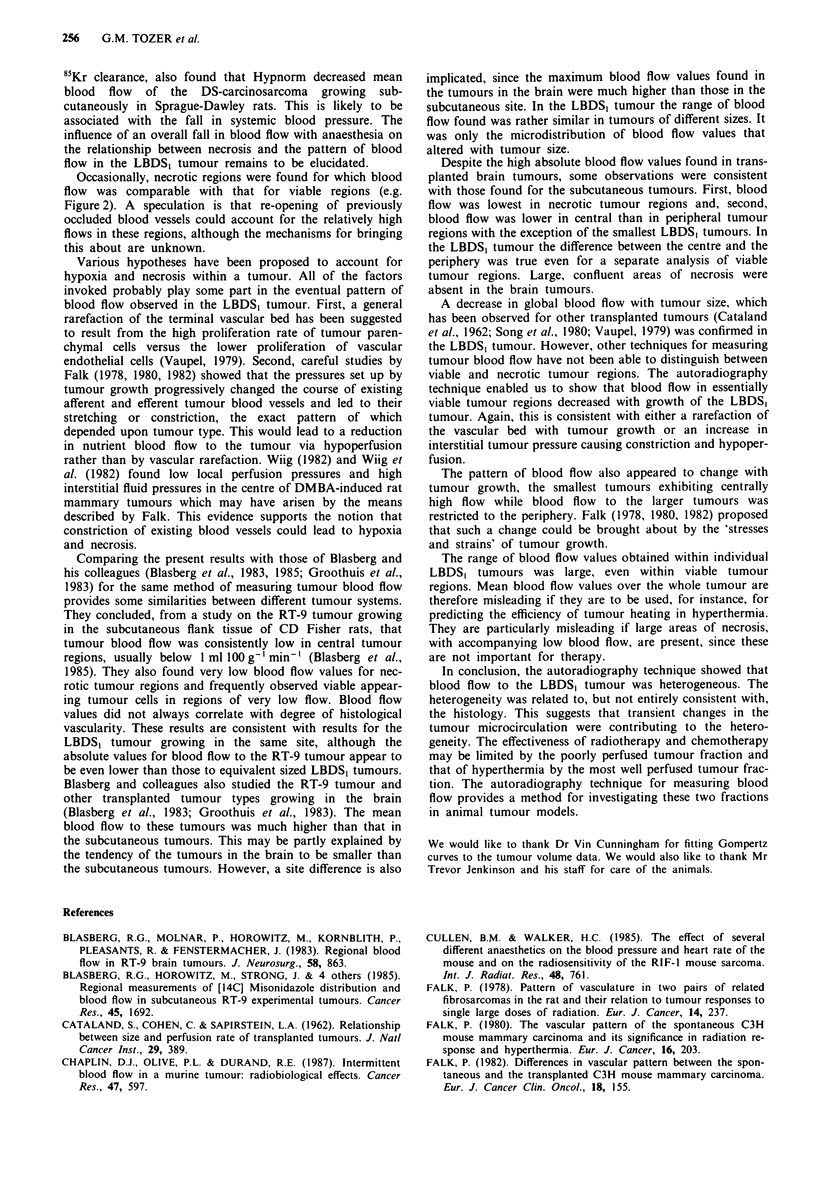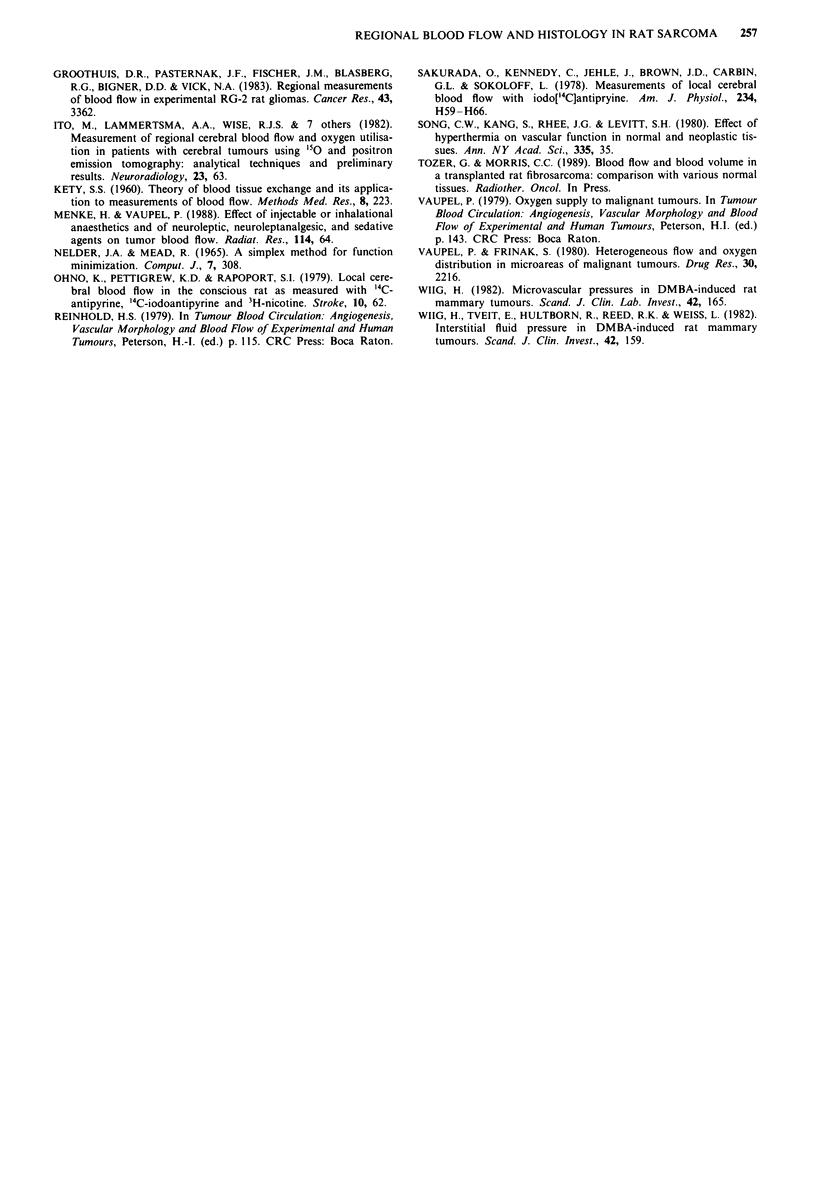# The relationship between regional variations in blood flow and histology in a transplanted rat fibrosarcoma.

**DOI:** 10.1038/bjc.1990.46

**Published:** 1990-02

**Authors:** G. M. Tozer, S. Lewis, A. Michalowski, V. Aber

**Affiliations:** Medical Research Council Cyclotron Unit, Hammersmith Hospital, London, UK.

## Abstract

**Images:**


Br. J. Cancer (1990), 61, 250-257
© Macmillan Press Ltd., 1990

### The relationship between regional variations in blood flow and histology in a transplanted rat fibrosarcoma

G.M. Tozer[1], S. Lewis[2], A. Michalowski[1] & V. Aber[2]

[1]*Medical Research Council Cyclotron Unit, Hammersmith Hospital; and* [2]*Department of Medical Physics, Royal Post-graduate Medical School, Hammersmith Hospital, DuCane Road, London W12 0HS, UK.*

**Summary** The regional distribution of blood flow to the $LBDS_1$ fibrosarcoma, transplanted into the subcutaneous site in rats, was investigated using the readily diffusible compound $^{14}$C-iodo-antipyrine ($^{14}$C-IAP). Quantitative autoradiography was used to establish absolute values of specific blood flow F for $100 \times 100 \times 20\,\mu m$ adjacent tissue volumes of the unperturbed tumour. Mean blood flow to whole tumours was found to decrease with increase in tumour size. This relationship was abolished if blood flow was only measured in sections cut from the periphery of the tumours. Detailed analysis of a sub-group of tumours showed that blood flow to individual tumours was heterogeneous. The range of blood flow was large, indicating that mean blood flow to a whole tumour is a poor reflection of the blood perfusion pattern of that tumour. Necrotic tumour regions were usually very poorly perfused. With the exception of the smallest tumours studied, blood flow was lower in the centre of tumours than in the periphery. Necrosis also tended to develop centrally. However, the peripheral to central gradient of blood flow was apparent even when densely cellular, viable tumour regions and necrotic regions were analysed separately. The decrease in blood flow with tumour size was also apparent in densely cellular, viable tumour regions when analysed separately. Qualitative comparison of tumour histology and regional blood flow showed that there were areas of very low blood flow associated with viable tumour regions. Less common were areas of rather high blood flow associated with necrotic tumour regions. A complicated relationship exists between tumour histology and blood flow. The quantitative autoradiography technique is suitable for investigating the most poorly perfused and the most well perfused viable fractions of animal tumours which may limit the efficacy of different types of therapy.

The blood flow to tumours plays an important role in their treatment. In radiotherapy, the efficacy of treatment depends upon cellular oxygen concentration which is governed by respiration rate and local blood flow. In chemotherapy, blood flow determines the efficiency of drug delivery. In hyperthermia, the temperature elevation achieved and the sensitivity of cells to heat is influenced by blood flow. Blood flow is therefore a critical parameter to measure both experimentally and clinically.

It is known that blood flow to both transplanted animal tumours and to human tumours is heterogeneous (Chaplin *et al.*, 1987; Ito *et al.*, 1982; Vaupel & Frinak, 1980). Therefore global measurements may not be the most pertinent means of describing tumour blood flow. For instance, global measurements will include blood flow to necrotic tumour regions which are not relevant for therapy. For hyperthermia, a knowledge of high blood flow in discrete tumour regions would be important because these regions are the ones most likely to limit efficacy of treatment.

Blasberg and colleagues used the inert, readily diffusible compound iodo-antipyrine to measure blood flow to transplanted rat brain tumours (Blasberg *et al.*, 1983; Groothuis *et al.*, 1983). Labelling of iodo-antipyrine with $^{14}$C ($^{14}$C-IAP) facilitated autoradiography of tumour slices so that the regional distribution of tumour blood flow could be investigated. The method had originally been developed by Sakurada *et al.* (1978) for measurement of regional blood flow in normal rat brains.

In the present study, $^{14}$C-IAP has been used to measure regional blood flow to unperturbed transplanted rat fibrosarcomas. Regional variations in blood flow were compared to regional variations in histology. It is known that global blood flow to transplanted tumours decreases with tumour growth (Cataland *et al.*, 1962; Song *et al.*, 1980; Vaupel, 1979). The influence of growth-related tumour necrosis on this relationship was investigated.

Correspondence: G.M. Tozer.
Received 20 July 1989; and in revised form 27 September 1989.

### Methods

### *Tumours*

A transplanted rat fibrosarcoma, designated $LBDS_1$, was used for these experiments. Details of the origin of this tumour and its maintenance have been given elsewhere (Tozer & Morris, 1989). Briefly, maintenance involves subcutaneous transplantation of 1–2 mm$^3$ tumour pieces into the right flanks of 8–12-week-old male BD9 rats. Only tumours between the seventh and fourteenth generations away from the original spontaneous tumour were used for these experiments.

Three orthogonal caliper measurements of tumour diameter were used to produce tumour growth curves. Tumour volume ($V$) was calculated using the formula:

$$V = \pi/_6\ (d_1 \times d_2 \times d_3)$$

where $d_1$, $d_2$ and $d_3$ are the orthogonal tumour diameters corrected for a double skin thickness of 1.5 mm.

Gompertz curves were fitted to the tumour volume data using the formula:

$$\ln V = a - be^{-c(t-d)}$$

where $a$, $b$ and $c$ are constants, $d$ is a constant representing the time between transplantation and start of tumour growth and $t$ is the time after transplantation.

Estimates of $a$, $b$, $c$ and $d$ were obtained from individual tumour plots of $\ln V$ against $t$, using a non-linear fitting routine which employed a simplex algorithm to minimise the residual sum of squares, with all points given equal weights (Nelder & Mead, 1965).

Tumour volume doubling times ($T_D$) were calculated at various times throughout tumour growth using the formula:

$$T_D = \frac{\ln 2}{bce^{-c(t-d)}}$$

### *Blood flow*

Blood flow was measured using the uptake, over a short infusion time, of the inert, readily diffusible compound, iodo-antipyrine. Sampling of arterial blood over the infusion time, measurement of tissue levels of iodo-antipyrine at the end of the infusion time and a knowledge of the relative solubility of iodo-antipyrine in tissue and blood allows calculation of the specific blood flow to a tissue F (Kety, 1960).

The details of the method for measuring blood flow using $^{14}C$-labelled iodo-antipyrine ($^{14}C$-IAP) have been published elsewhere (Tozer & Morris, 1989). In that publication tissue levels of $^{14}C$-IAP were measured using liquid scintillation. In the present study tissue activity levels were measured using autoradiography of frozen tissue sections.

Briefly, tumour-bearing rats were anaesthetised with fentanyl citrate (0.315 mg kg$^{-1}$) and fluanisone (10 mg kg$^{-1}$) ('Hypnorm', Crown Chemical Co. Ltd) and midazolam (5 mg kg$^{-1}$) ('Hypnovel', Roche). Polyethylene catheters containing heparinised 0.9% phosphate buffered saline were implanted into the right carotid artery, jugular vein and left femoral vein. $^{14}C$-IAP (Amersham), 1.11 MBq (30 $\mu$Ci) in 0.4 ml 0.9% phosphate buffered saline, was infused into the rat's circulation via the femoral vein catheter over a 30s period. Arterial blood samples, in the form of free-flowing blood from the carotid catheter, were taken every second over the 30s infusion period (approximately 0.05 g s$^{-1}$). Throughout this procedure rectal temperature was maintained at 35.0–37.5°C using a thermostatically controlled heating blanket. At the end of the 30s period the rat was killed and blood flow terminated by bolus injection of 0.3 ml saturated KCl via the jugular vein catheter. Tumours were removed rapidly from the dead animal and frozen in isopentane at −40 to −50°C. They were stored at −70°C until the assay time and orientated with respect to their position in the animal (see Figure 1). $^{14}C$ activity in weighed arterial blood samples ($C_a$) was measured using liquid scintillation counting. Each blood sample was dissolved in 1 ml Soluene-350 (Packard) and left overnight. They were bleached with 0.5 ml 30% hydrogen peroxide and counted using Dimilume (Packard) as the scintillant and suitable quench correction.

### *Autoradiography*

Tissue activity levels are measured using autoradiography. Cryostat sections 20 $\mu$m thick were cut perpendicular to the skin surface from the periphery of the tumour to the centre in 1 mm steps as shown in Figure 1. Two sections were cut for autoradiography and two sections were cut for histology at each 1 mm step. Sections for autoradiography were picked up onto warm coverslips and sections for histology were picked up onto warm microscope slides. Both types were dried rapidly on a hot plate set at 60°C. Sections for histology were fixed in formal saline and stained with haematoxylin and eosin. The coverslips were mounted onto cardboard sheets together with methyl methacrylate standards of known $^{14}C$ activity (Amersham). Under dark room conditions, autoradiographic film (Hyperfilm-$\beta$max, Amersham) was overlaid onto the tissue sections which were then stored in metal cassettes. Films were exposed for 21 days and developed following the instructions supplied with the film.

Grey levels, corrected for background, were measured for each tissue section and each plastic standard, at 100 $\mu$m intervals, using a scanning densitometer (Chromoscan, Joyce Loebl). Results were transferred onto a Perkin-Elmer 32/30 computer for analysis.

**Figure 1** Rat bearing subcutaneous tumour showing the orientation of cryostat tumour sections with respect to the animal. The parallel planes show the direction in which the cryostat sections were cut from the excised tumours. The arrow indicates the order in which sections were cut from the periphery towards the centre of each tumour. The expanded section shows how each section was labelled with respect to its original position.

### *Mathematical analysis*

Blood flow was calculated using the equation derived by Kety (1960):

$$C(T) = mF \int_0^T C_a e^{\frac{-mF(T-t)}{\lambda}} dt \qquad (1)$$

where $F$ is specific blood flow; $C(T)$ is tissue concentration of $^{14}C$-IAP, measured by autoradiography, at time $t = T$, the end of the infusion time; $C_a$ is arterial concentration of $^{14}C$-IAP, measured by liquid scintillation, from time $t = 0$ (when the arterial concentration starts to rise) to time $t = T$; $\lambda$ is tissue–blood partition coefficient of $^{14}C$-IAP; $m$ is a value between 0 and 1 reflecting the extent to which diffusional equilibrium is established between tissue and blood.

In these experiments $m$ was assumed to be 1, representing diffusional equilibrium. A value of 0.8 was used for $\lambda$ which has previously been established for these tumours (Tozer & Morris, 1989).

A double exponential curve was fitted to the relationship between arterial blood $^{14}C$-IAP concentration ($C_a$) and time ($t$) following the method of Ohno *et al.* (1979) where:

$$C_a = A + Be^{-Rt} + De^{-St} \qquad (2)$$

where $A$, $B$, $D$, $R$ and $S$ are constants.

Ingegration of equation (2) and substituting into equation (1) gives the working form of the Kety equation quoted by Ohno *et al.* (1979):

$$\frac{C(T)}{\lambda} = A - ((A + Bk)/(k - R) + Dk/(k - S))e^{-kT} + (k(k - R)\ Be^{-Rt} + (k/(k - S))\ De^{-St} \quad (3)$$

where $k = \frac{mF}{\lambda}$

Values for $C(T)$ were determined for each possible grey level from 0 to 255 in each tumour by calibration of tissue grey levels obtained from the autoradiograms of plastic standards of known radioactivity. Values for $C(T)$ therefore represented intraparenchymal plus intravascular tissue activities.

Equation (3) was solved for $F$ for each possible value of $C(T)$, in each tumour, using an iterative procedure on the Perkin-Elmer 32/30 computer. Thus, a value for $F$ could be allocated to each of the densitometer-defined, adjacent $100 \times 10 \times 20\,\mu m$ tissue volumes in a tumour section. This amounted to approximately 48,000 values for $F$ for the largest sections studied. Regions of interest in each section were defined as described below and results for each region of interest were expressed as histograms, for which the number of $100 \times 100 \times 20\,\mu m$ tissue volumes corresponding to each value of $F$ was evaluated. A mean value for $F$ in each region of interest was also calculated.

### *Image analysis*

Computed pseudo-colour images of blood flow data ($F$ values) were produced on a Tektronix 4207 colour graphics terminal for each tumour section for comparison with the corresponding histology. The Ghost-80 graphics library was utilised for image processing.

Grey levels from the scanning densitometer were digitised to 256 levels of density and transformed into $F$ values as described in the previous section. The 256 levels were reduced to 16 for display purposes, using a linear transformation. Each display level was allocated a different colour using a 'colour palette' table of hue, lightness and shading contained in a data file on the computer.

Blood flow data were displayed by examining each scan line in turn and transforming each data point into the appropriate display level. This was facilitated by using a system of 'run encoding' where advantage is taken of the likelihood that adjacent data values fall into the same display colour class. Where this condition holds true a coloured line can be drawn on the screen linking the first and last data point of the same colour class. This decreases the amount of information that must be sent to the graphics terminal since the positional information need only be sent for two points, rather than for all points in the same colour class of a particular region. A colour wedge relating display colours to blood flow was included in the final display.

For comparison of histology and blood flow, images of histological sections were projected onto plastic sheets such that the size exactly matched the size of the appropriate image of computed blood flow. Regions of different histologies, for each tumour section, were delineated on the plastic sheets. The sheets were overlaid onto the computed blood flow images and the outlines of the regions transferred onto the blood flow image using a 'mouse'. A mean blood flow value and a histogram showing the range of blood flow values were obtained for each delineated region as well as for each section as a whole. Boundaries were marked by drawing a series of straight lines around the selected area. The data contained within each area were written to a file for subsequent analysis. Up to 10 areas could be selected at any one time.

### Results

Microscopic examination of tumour sections revealed the tumours to consist of irregularly accumulated cells with round or oval nuclei and ill-defined cell outlines. The cells were ordinarily densely packed ($10.6 \pm 0.9 \times 10^3$ cell nuclei per $mm^2$) without distinct deposits of extracellular material, the overall picture corresponding to poorly differentiated sarcoma. All tumours showed a network of capillaries, and some included a few arterioles and venules, as well as remnants of a thin connective tissue capsule. Larger tumours displayed irregular areas of coagulative necrosis varying in size. In addition, some large tumours included well delineated oedematous areas containing a low density of viable cells ($2.3 \pm 0.7 \times 10^3$ cell nuclei per $mm^2$). These areas could represent the final outcome of focal necrosis locally affecting a majority of cells which have disintegrated and become resorbed.

Based on this description, tumour histology was divided into three distinct categories for further analysis. These were viable densely cellular tissue, viable sparsely cellular tissue and necrotic tissue. As examples, Figure 2 shows diagrammatic versions of two tumours (volumes $752\,mm^3$ and $4{,}861\,mm^3$) where the histology has been divided into these categories. Figure 3 shows the corresponding computed blood flow images for the two tumours. The sections were taken from the centre of each tumour. The numbers in the histology diagrams show the mean blood flow for each tumour region. Using this method, blood flow was compared to its corresponding histology for nine different tumours. Qualitatively, this revealed that: (1) Tumours showing a uniform, viable, densely cellular pattern of histology still show a non-uniform distribution of blood flow (e.g. $752\,mm^3$ tumour). Occasionally, the presence of small arteries in the vicinity of the most well perfused regions could have partially accounted for this heterogeneity. This was not the case for the tumours shown in Figures 2 and 3. (2) Blood flow to necrotic regions is low. However, these low blood flow areas extend well beyond the histologically defined necrotic regions adjacent to the grossly necrotic regions. (3) Well perfused tumour regions tend to be around the tumour periphery (e.g. $4{,}861\,mm^3$ tumour). However, there are no orientation effects with respect to rostral or caudal tumour regions or regions adjacent to the skin or the body wall. (4) Well perfused tumour regions tend to be associated with viable, densely cellular tumour regions. However, a minority of necrotic tumour regions are well perfused (e.g. there are three necrotic regions in the $4{,}861\,mm^3$ tumour in Figure 2 with blood flow $> 10\,ml\,100\,g^{-1}\,min^{-1}$).

Additional information was obtained from quantitative analyses. Figure 4 shows the pattern of growth of the $LBDS_1$ tumour during its measurable phase. The doubling times show a progressive slowing of growth with increasing tumour volume. Means $\pm$ s.d. for $a$, $b$, $c$ and $d$ for the group of tumours shown in Figure 4 were found to be $9.61 \pm 0.88$, $9.08 \pm 4.0$, $0.17 \pm 0.06$ and $25.04 \pm 2.38$ respectively.

Figure 5 shows that an overall decrease in tumour blood flow accompanies the slowing of tumour growth with increasing tumour size (panel a). The data fit the relationship $\log F = 1.54 - 0.101 \log V$ ($0.001 < P < 0.01$). This is similar to results obtained by others for different tumours and different methods of measuring blood flow (Cataland *et al*., 1962; Reinhold, 1979; Song *et al*., 1980). Figure 5b shows that a similar but slightly clearer relationship exists between blood flow and tumour size when blood flow is only measured from the sections taken from the centre of tumours ($\log F = 1.61 - 0.139 \log V$). This is despite the fact that these 'central' sections inevitably include peripheral tumour regions around their edges. Figure 5c shows that this relationship is entirely lost if blood flow is only measured from sections taken from the periphery of the tumours.

These results could be explained purely on the basis of large tumours developing extensive areas of central necrosis which are poorly perfused compared to the periphery. In order to investigate this, further detailed analyses were carried out on nine of the tumours whose volumes ranged from 16 to $4{,}861\,mm^3$. Two tumour sections, one from the periphery and one from the centre of each tumour, were chosen for histological analysis together with the adjacent autoradiograms. Blood flows to histologically defined regions in these sections were established as described in the previous section.

Figure 6 shows mean blood flow values obtained for each section as a whole (panel a) and the mean blood flow values obtained from densely cellular regions (panel b) and necrotic regions (panel c) of each section. The results are sub-divided into sections taken from the periphery and from the centre of

**Figure 2** Diagrammatic representation of tumour histology for 752 $mm^3$ tumour (**a**) and 4,861 $mm^3$ tumour (**b**). Corresponding blood flow is shown in Figure 3. ☐, viable densely cellular tissue; ☐, viable sparsely cellular tissue; ☐, necrotic tissue. Numbers represent the mean blood flow in ml 100 $g^{-1}$ $min^{-1}$ for each tumour region.

**Figure 3** Computed image of tumour blood flow for 752 $mm^3$ tumour (**a**) and 4,861 $mm^3$ tumour (**b**). Corresponding histology is shown in Figure 2.

the tumours. Each pair of columns represents a single tumour and they are ranked from left to right in size order. Figure 6a shows that this sub-group of tumours conforms to the pattern shown for the whole group in Figure 5a and b. Tumour blood flow is higher in the periphery than in the centre for all tumours over 800 $mm^3$ and the decrease in blood flow with tumour size is apparent only for central sections. Blood flow is actually higher in the centre than in the periphery for the two smallest tumours ($<700$ $mm^3$). Figure 6b shows that reduced blood flow in the central sections is also apparent for all but one of the six largest tumours if data from dense regions only are analysed. Blood flow to dense regions of central sections showed the same pattern of a decrease with tumour size as in Figure 6a. Figure 6c shows that there is also a decrease in blood flow to necrotic tumour regions towards the centre of the tumours. In this case the four smallest tumours were excluded from the analysis since most of the sections from these tumours contained no necrotic regions. The largest amount of necrosis in any one of the sections from these tumours only contained 1,539 values for $F$. In the tumours which were analysed in Figure 6c there were at least 5,000 values for $F$ in necrotic regions of any one section. The analysis showed that there was no relationship between blood flow and tumour size in necrotic regions.

Summarising Figure 6, blood flow in central tumour regions tends to be higher than in the periphery for the smallest tumours analysed. This situation is reversed for larger tumours and those which have significant levels of necrosis. In the significantly necrotic tumours, the decrease in

**Figure 4** Growth of the $LBDS_1$ tumour with time after transplantation. Lines show fitted Gompertz curves to individual tumour data. Actual tumour volumes are shown for one tumour. Data for the other tumours have been omitted for clarity. The inset shows an increase in mean volume doubling time for the seven tumours with increasing tumour size.

**Figure 5** Blood flow to the $LBDS_1$ tumour versus tumour volume. Each point in **a** represents a mean value of blood flow for individual tumours. The mean value was obtained from all the individual values of *F* which represent blood flow to $100 \times 100 \times 20\,\mu m$ tissue volumes. Values for *F* from sections cut throughout the whole tumour were used. Each point in **b** also represents a mean value of blood flow from individual values of *F*, but only values of *F* from the section positioned most centrally in each tumour were used. Each point in **c** also represents a mean value of blood flow from individual values of *F*, but only values of *F* from the two sections positioned most peripherally in each tumour were used.

blood flow towards the centre of the tumour is not entirely due to a larger contribution from necrotic areas in the centre of tumours *per se*, since a substantial difference between central and peripheral blood flow also exists within both viable, densely cellular tumour regions and necrotic tumour regions. Similarly, the decline in blood flow with tumour size for central sections is not entirely due to an increase in necrotic fraction with tumour size since the change in blood flow with tumour size in the densely cellular regions alone shows the same pattern.

Table I shows the range of blood flow values obtained for the nine tumours shown in Figure 6 together with mean blood flow values for each section shown in parentheses. This demonstrates that, unlike the mean blood flow values, the range of blood flow (*F*) values obtained does not decrease towards the centre of the tumour or with tumour growth. It also demonstrates that use of a mean blood flow value for a particular section, although useful, obscures the presence of, on the one hand, very poorly perfused tumour regions and, on the other hand, efficiently perfused regions.

Figures 7 and 8 show examples of histograms obtained from the two tumours shown in Figures 2 and 3, with volumes of 752 $mm^3$ and 4,861 $mm^3$ respectively. The smaller tumour has only a small necrotic region, so that only data from viable, densely cellular regions are shown in Figure 7. The larger tumour (Figure 8) has been sub-divided into viable densely cellular, viable, sparsely cellular and necrotic regions. Figure 7 shows that the range of blood flow values obtained for the peripheral and central sections of the smaller tumour are very similar and there are no obvious differences between the distributions of blood flow within this range. Figure 8 shows that, despite a similar range of blood flow in the peripheral and central section of the larger tumour, the decline in blood flow towards the centre is due to an increase in the proportion of low blood flow readings in all three histological categories.

### Discussion

The heterogeneous distribution of blood flow in the $LBDS_1$ fibrosarcoma probably results from both short-term alterations in tumour blood flow and a longer term response of the tumour vasculature to tumour growth. Evidence for the former comes from studies of tumours growing in transparent chambers (e.g. Reinhold, 1979) and from the work of Chaplin *et al.* (1987) using the fluorescent dye Hoechst 33342. This property of intermittent flow in transplanted tumour blood vessels could explain the heterogeneity of blood flow within histologically well defined, densely cellular regions of the $LBDS_1$ tumour. It could also explain why blood flow values did not always correlate with histologically visible vascularity. Despite the heterogeneity of blood flow within histologically distinct regions there was still a clear overall relationship between the tumour histology and its blood flow, indicating a longer term change in tumour vascularisation. Most necrotic tumour regions received very little, if any, blood flow. However, these very low blood flow regions extended well beyond the necrotic regions into viable, sparsely or densely cellular regions. This implies a decreasing

**Figure 6** Blood flow to nine examples of the $LBDS_1$ tumour ranked in order of increasing tumour volume from left to right along the horizontal axis. A mean blood flow value is calculated for each tumour from individual values of *F* determined from either the section positioned most centrally in each tumour (solid bars) or the section positioned most peripherally in each tumour (hatched bars). **a** represents *F* values from whole sections covering all histological categories. **b** represents *F* values from histologically viable, densely cellular regions only. **c** represents *F* values from histologically necrotic regions only.

blood flow from the arterial end of the tumour circulation to the terminal vascular bed which eventually results in necrosis. Presumably the cells in the viable, sparsely or densely cellular regions where the blood flow is very low have not been subjected to these low flows for sufficient time to produce overt necrosis. Such low blood flow values do imply, however, that radiobiologically hypoxic, viable cells exist in these tumours.

The effect of Hypnorm and midazolam anaesthesia on the blood flow pattern of the $LBDS_1$ tumour is not known. This mixture, in common with other injectable anaesthetics, is known to reduce systemic blood pressure in rodents (Cullen & Walker, 1985). Hypnorm alone also has this effect (Menke & Vaupel, 1988). We have found similar reductions in systemic blood pressure for the BD9 rat following Hypnorm and midazolam anaesthesia. Menke & Vaupel (1988), using

**Table I**

| | | *Blood flow (ml 100 g⁻¹ min⁻¹)*[a] | | | | | |
|---|---|---|---|---|---|---|---|
| | | *Peripheral* | | | *Central* | | |
| *Tumour* | *Tumour volume (mm³)* | *Viable densely cellular* | *Viable sparsely cellular* | *Necrotic* | *Viable densely cellular* | *Viable sparsely cellular* | *Necrotic* |
| 1 | 16 | 0–24.1 (11.9) | – | – | 0–46.2 (19.7) | – | – |
| 2 | 395 | 0–51.3 (15.0) | – | – | 0–50.6 (18.6) | – | 0–8.9 (4.7) |
| 3 | 752 | 0–51.3 (16.8) | – | – | 0–61.2 (16.8) | – | 0–19.1 (4.2) |
| 4 | 928 | 0–59.1 (19.2) | – | – | 0–70.4 (9.9) | – | – |
| 5 | 2302 | 0–49.2 (8.9) | 0–49.8 (3.8) | 0–35.8 (3.8) | 0–78.4 (6.6) | 0–104.1 (17.8) | 0–20.4 (1.8) |
| 6 | 2302 | 0–75.9 (17.5) | – | 0–9.6 (4.3) | 0–76.8 (9.8) | – | 0–24.0 (2.9) |
| 7 | 3057 | 0–38.2 (11.7) | – | 0–12.0 (4.4) | 0–36.4 (6.1) | 0–20.7 (5.2) | 0–37.1 (3.9) |
| 8 | 3356 | 0–36.3 (6.1) | 0–32.1 (6.4) | 0–14.3 (4.1) | 0–76.8 (7.3) | 0–39.5 (4.6) | 0–11.5 (3.2) |
| 9 | 4861 | 0–67.7 (15.9) | 0–58.0 (8.2) | 0–20.1 (6.7) | 0–65.0 (8.5) | 0–20.1 (2.7) | 0–54.0 (2.9) |

[a]Blood flow values are the range of F values obtained for each section (min. to max.) with the mean value for each section shown in parentheses.

**Figure 7** The range of blood flow found in a central section and a peripheral section of a 752 mm³ tumour. The vertical axis is the % *F* values calculated for individual 100 × 100 × 20 µm tumour volumes which fall into the blood flow categories shown on the horizontal axis. Data are for histologically viable, densely cellular tumour regions only. There were no sparsely cellular regions present in this tumour. The necrotic region (see Figure 2) was too small to include in the analysis.

**Figure 8** The range of blood flow found in a central section and a peripheral section of a 4,861 mm³ tumour. The vertical axis is the % values for *F* calculated for individual 100 × 100 × 20 µm tumour volumes which fall into the blood flow categories shown on the horizontal axes. The tumour is sub-divided into histologically viable densely cellular (■), viable sparsely cellular (▨) and necrotic (▤) regions.

$^{85}$Kr clearance, also found that Hypnorm decreased mean blood flow of the DS-carcinosarcoma growing subcutaneously in Sprague-Dawley rats. This is likely to be associated with the fall in systemic blood pressure. The influence of an overall fall in blood flow with anaesthesia on the relationship between necrosis and the pattern of blood flow in the $LBDS_1$ tumour remains to be elucidated.

Occasionally, necrotic regions were found for which blood flow was comparable with that for viable regions (e.g. Figure 2). A speculation is that re-opening of previously occluded blood vessels could account for the relatively high flows in these regions, although the mechanisms for bringing this about are unknown.

Various hypotheses have been proposed to account for hypoxia and necrosis within a tumour. All of the factors invoked probably play some part in the eventual pattern of blood flow observed in the $LBDS_1$ tumour. First, a general rarefaction of the terminal vascular bed has been suggested to result from the high proliferation rate of tumour parenchymal cells versus the lower proliferation of vascular endothelial cells (Vaupel, 1979). Second, careful studies by Falk (1978, 1980, 1982) showed that the pressures set up by tumour growth progressively changed the course of existing afferent and efferent tumour blood vessels and led to their stretching or constriction, the exact pattern of which depended upon tumour type. This would lead to a reduction in nutrient blood flow to the tumour via hypoperfusion rather than by vascular rarefaction. Wiig (1982) and Wiig *et al.* (1982) found low local perfusion pressures and high interstitial fluid pressures in the centre of DMBA-induced rat mammary tumours which may have arisen by the means described by Falk. This evidence supports the notion that constriction of existing blood vessels could lead to hypoxia and necrosis.

Comparing the present results with those of Blasberg and his colleagues (Blasberg *et al.*, 1983, 1985; Groothuis *et al.*, 1983) for the same method of measuring tumour blood flow provides some similarities between different tumour systems. They concluded, from a study on the RT-9 tumour growing in the subcutaneous flank tissue of CD Fisher rats, that tumour blood flow was consistently low in central tumour regions, usually below $1\ ml\ 100\ g^{-1}\ min^{-1}$ (Blasberg *et al.*, 1985). They also found very low blood flow values for necrotic tumour regions and frequently observed viable appearing tumour cells in regions of very low flow. Blood flow values did not always correlate with degree of histological vascularity. These results are consistent with results for the $LBDS_1$ tumour growing in the same site, although the absolute values for blood flow to the RT-9 tumour appear to be even lower than those to equivalent sized $LBDS_1$ tumours. Blasberg and colleagues also studied the RT-9 tumour and other transplanted tumour types growing in the brain (Blasberg *et al.*, 1983; Groothuis *et al.*, 1983). The mean blood flow to these tumours was much higher than that in the subcutaneous tumours. This may be partly explained by the tendency of the tumours in the brain to be smaller than the subcutaneous tumours. However, a site difference is also implicated, since the maximum blood flow values found in the tumours in the brain were much higher than those in the subcutaneous site. In the $LBDS_1$ tumour the range of blood flow found was rather similar in tumours of different sizes. It was only the microdistribution of blood flow values that altered with tumour size.

Despite the high absolute blood flow values found in transplanted brain tumours, some observations were consistent with those found for the subcutaneous tumours. First, blood flow was lowest in necrotic tumour regions and, second, blood flow was lower in central than in peripheral tumour regions with the exception of the smallest $LBDS_1$ tumours. In the $LBDS_1$ tumour the difference between the centre and the periphery was true even for a separate analysis of viable tumour regions. Large, confluent areas of necrosis were absent in the brain tumours.

A decrease in global blood flow with tumour size, which has been observed for other transplanted tumours (Cataland *et al.*, 1962; Song *et al.*, 1980; Vaupel, 1979) was confirmed in the $LBDS_1$ tumour. However, other techniques for measuring tumour blood flow have not been able to distinguish between viable and necrotic tumour regions. The autoradiography technique enabled us to show that blood flow in essentially viable tumour regions decreased with growth of the $LBDS_1$ tumour. Again, this is consistent with either a rarefaction of the vascular bed with tumour growth or an increase in interstitial tumour pressure causing constriction and hypoperfusion.

The pattern of blood flow also appeared to change with tumour growth, the smallest tumours exhibiting centrally high flow while blood flow to the larger tumours was restricted to the periphery. Falk (1978, 1980, 1982) proposed that such a change could be brought about by the 'stresses and strains' of tumour growth.

The range of blood flow values obtained within individual $LBDS_1$ tumours was large, even within viable tumour regions. Mean blood flow values over the whole tumour are therefore misleading if they are to be used, for instance, for predicting the efficiency of tumour heating in hyperthermia. They are particularly misleading if large areas of necrosis, with accompanying low blood flow, are present, since these are not important for therapy.

In conclusion, the autoradiography technique showed that blood flow to the $LBDS_1$ tumour was heterogeneous. The heterogeneity was related to, but not entirely consistent with, the histology. This suggests that transient changes in the tumour microcirculation were contributing to the heterogeneity. The effectiveness of radiotherapy and chemotherapy may be limited by the poorly perfused tumour fraction and that of hyperthermia by the most well perfused tumour fraction. The autoradiography technique for measuring blood flow provides a method for investigating these two fractions in animal tumour models.

We would like to thank Dr Vin Cunningham for fitting Gompertz curves to the tumour volume data. We would also like to thank Mr Trevor Jenkinson and his staff for care of the animals.

### References

BLASBERG, R.G., MOLNAR, P., HOROWITZ, M., KORNBLITH, P., PLEASANTS, R. & FENSTERMACHER, J. (1983). Regional blood flow in RT-9 brain tumours. *J. Neurosurg.*, **58**, 863.

BLASBERG, R.G., HOROWITZ, M., STRONG, J. & 4 others (1985). Regional measurements of [14C] Misonidazole distribution and blood flow in subcutaneous RT-9 experimental tumours. *Cancer Res.*, **45**, 1692.

CATALAND, S., COHEN, C. & SAPIRSTEIN, L.A. (1962). Relationship between size and perfusion rate of transplanted tumours. *J. Natl Cancer Inst.*, **29**, 389.

CHAPLIN, D.J., OLIVE, P.L. & DURAND, R.E. (1987). Intermittent blood flow in a murine tumour: radiobiological effects. *Cancer Res.*, **47**, 597.

CULLEN, B.M. & WALKER, H.C. (1985). The effect of several different anaesthetics on the blood pressure and heart rate of the mouse and on the radiosensitivity of the RIF-1 mouse sarcoma. *Int. J. Radiat. Res.*, **48**, 761.

FALK, P. (1978). Pattern of vasculature in two pairs of related fibrosarcomas in the rat and their relation to tumour responses to single large doses of radiation. *Eur. J. Cancer*, **14**, 237.

FALK, P. (1980). The vascular pattern of the spontaneous C3H mouse mammary carcinoma and its significance in radiation response and hyperthermia. *Eur. J. Cancer*, **16**, 203.

FALK, P. (1982). Differences in vascular pattern between the spontaneous and the transplanted C3H mouse mammary carcinoma. *Eur. J. Cancer Clin. Oncol.*, **18**, 155.

GROOTHUIS, D.R., PASTERNAK, J.F., FISCHER, J.M., BLASBERG, R.G., BIGNER, D.D. & VICK, N.A. (1983). Regional measurements of blood flow in experimental RG-2 rat gliomas. *Cancer Res.*, **43**, 3362.

ITO, M., LAMMERTSMA, A.A., WISE, R.J.S. & 7 others (1982). Measurement of regional cerebral blood flow and oxygen utilisation in patients with cerebral tumours using $^{15}O$ and positron emission tomography: analytical techniques and preliminary results. *Neuroradiology*, **23**, 63.

KETY, S.S. (1960). Theory of blood tissue exchange and its application to measurements of blood flow. *Methods Med. Res.*, **8**, 223.

MENKE, H. & VAUPEL, P. (1988). Effect of injectable or inhalational anaesthetics and of neuroleptic, neuroleptanalgesic, and sedative agents on tumor blood flow. *Radiat. Res.*, **114**, 64.

NELDER, J.A. & MEAD, R. (1965). A simplex method for function minimization. *Comput. J.*, **7**, 308.

OHNO, K., PETTIGREW, K.D. & RAPOPORT, S.I. (1979). Local cerebral blood flow in the conscious rat as measured with $^{14}C$-antipyrine, $^{14}C$-iodoantipyrine and $^{3}H$-nicotine. *Stroke*, **10**, 62.

REINHOLD, H.S. (1979). In *Tumour Blood Circulation: Angiogenesis, Vascular Morphology and Blood Flow of Experimental and Human Tumours*, Peterson, H.-I. (ed.) p. 115. CRC Press: Boca Raton.

SAKURADA, O., KENNEDY, C., JEHLE, J., BROWN, J.D., CARBIN, G.L. & SOKOLOFF, L. (1978). Measurements of local cerebral blood flow with iodo[$^{14}C$]antipryine. *Am. J. Physiol.*, **234**, H59–H66.

SONG, C.W., KANG, S., RHEE, J.G. & LEVITT, S.H. (1980). Effect of hyperthermia on vascular function in normal and neoplastic tissues. *Ann. NY Acad. Sci.*, **335**, 35.

TOZER, G. & MORRIS, C.C. (1989). Blood flow and blood volume in a transplanted rat fibrosarcoma: comparison with various normal tissues. *Radiother. Oncol.* In Press.

VAUPEL, P. (1979). Oxygen supply to malignant tumours. In *Tumour Blood Circulation: Angiogenesis, Vascular Morphology and Blood Flow of Experimental and Human Tumours*, Peterson, H.I. (ed.) p. 143. CRC Press: Boca Raton.

VAUPEL, P. & FRINAK, S. (1980). Heterogeneous flow and oxygen distribution in microareas of malignant tumours. *Drug Res.*, **30**, 2216.

WIIG, H. (1982). Microvascular pressures in DMBA-induced rat mammary tumours. *Scand. J. Clin. Lab. Invest.*, **42**, 165.

WIIG, H., TVEIT, E., HULTBORN, R., REED, R.K. & WEISS, L. (1982). Interstitial fluid pressure in DMBA-induced rat mammary tumours. *Scand. J. Clin. Invest.*, **42**, 159.