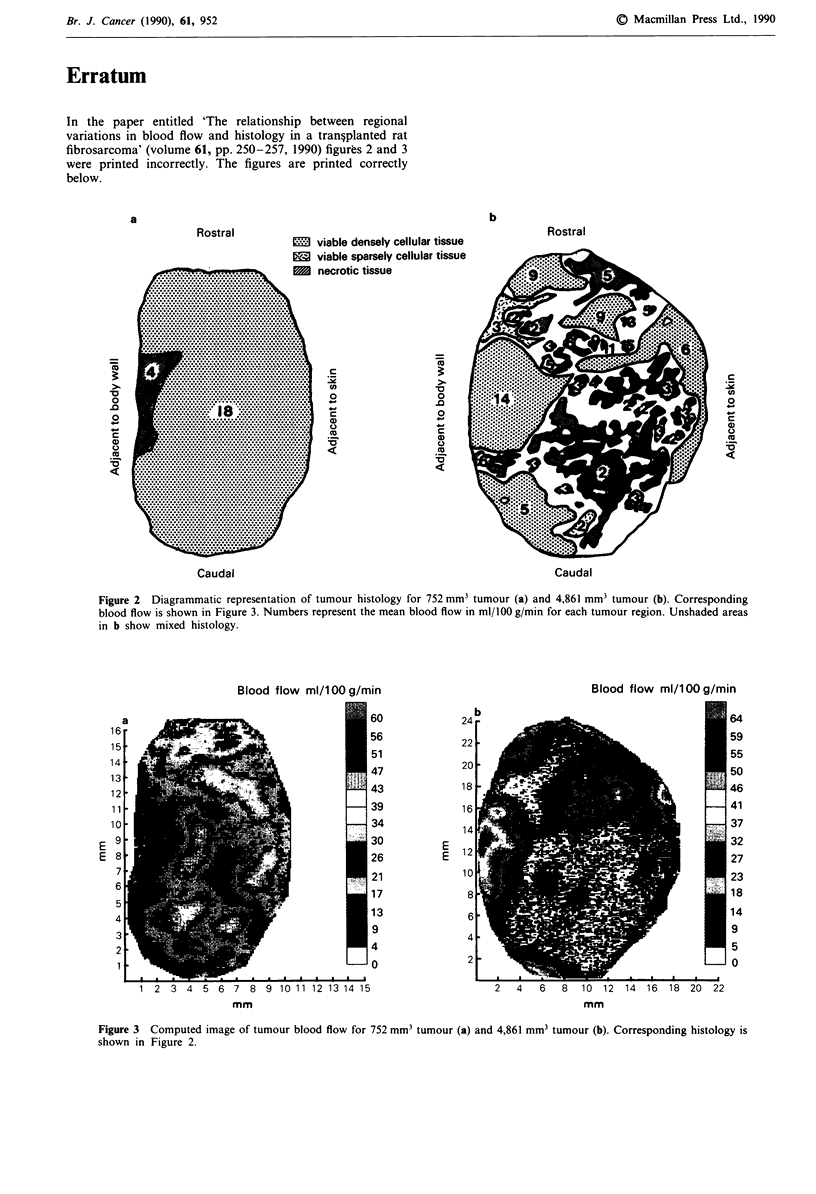# Erratum

**Published:** 1990-06

**Authors:** 

## Abstract

**Images:**


					
Br. J. Cancer (1990), 61, 952                                                                     ?  Macmillan Press Ltd., 1990

Erratum

In the paper entitled 'The relationship between regional
variations in blood flow and histology in a tran?planted rat
fibrosarcoma' (volume 61, pp. 250-257, 1990) figures 2 and 3
were printed incorrectly. The figures are printed correctly
below.

a

Rostral

b

viable densely cellular tissue
viable sparsely cellular tissue
necrotic tissue

C
.0

0

4-

C)

.)

IL
CU

Rostral

-o
0

-o
0

4-

C)
01

(i
co

Caudal

C
U)
0

C)

.X

U

(U

Caudal

Figure 2 Diagrammatic representation of tumour histology for 752 mm3 tumour (a) and 4,861 mm3 tumour (b). Corresponding
blood flow is shown in Figure 3. Numbers represent the mean blood flow in ml/100 g/min for each tumour region. Unshaded areas
in b show mixed histology.

flow ml/100g/min

60
56
51

i          ~~~43

39
34
21

I          ~~~1 3

9
4
0

b
24

22
20
18
16

14.
E 12W

10 11 12 13 14 15

mm

Blood flow ml/100 g/min

_              ~~~~~~~~~64

~~~~~~~~~~~~~~~~~~~5 ct

*3i~~~~~~~~3

*            114~~~~~~~3

6  8 lo 12  4  6  1  2  2

mm

Figure 3 Computed image of tumour blood flow for 752 mm3 tumour (a) and 4,861 mm3 tumour (b). Corresponding histology is

shown in Figure 2.

3

V
0
0

.0

4-0

CZ

C.)

(a

i6

Blood

16
15
14
13
12
1 1
10

E9

E8

4
9
5
0
6
1
7
2
7
3
3
4

3
2
1

1-   a   .               -     -

1  2  3  4   5  6  7  8  9